# Impact of different diagnostic technologies for MRSA admission screening in hospitals – a decision tree analysis

**DOI:** 10.1186/s13756-015-0093-0

**Published:** 2015-12-03

**Authors:** Claudia Hübner, Nils-Olaf Hübner, Christian Wegner, Steffen Flessa

**Affiliations:** Institute of Health Care Management, University of Greifswald, Friedrich-Loeffler-Str. 70, 17489 Greifswald, Germany; Institute of Hygiene and Environmental Health, University Medicine of Greifswald, Greifswald, Germany; IMD laboratory network, MVZ Greifswald GmbH, Greifswald, Germany

**Keywords:** Admission screening, Point-of-care, Hospital, Cost, Decision tree analysis, Methicillin-resistant Staphylococcus aureus

## Abstract

**Background:**

Hospital infections with multiresistant bacteria, e.g., Methicillin-resistant *Staphylococcus aureus* (MRSA), cause heavy financial burden worldwide. Rapid and precise identification of MRSA carriage in combination with targeted hygienic management are proven to be effective but incur relevant extra costs. Therefore, health care providers have to decide which MRSA screening strategy and which diagnostic technology should be applied according to economic criteria.

**Aim:**

The aim of this study was to determine which MRSA admission screening and infection control management strategy causes the lowest expected cost for a hospital. Focus was set on the Point-of-Care Testing (PoC).

**Methods:**

A decision tree analytic cost model was developed, primarily based on data from peer-reviewed literature. In addition, univariate sensitivity analyses of the different input parameters were conducted to study the robustness of the results.

**Findings:**

In the basic analysis, risk-based PoC screening showed the highest mean cost savings with 14.98 € per admission in comparison to no screening. Rapid universal screening methods became favorable at high MRSA prevalence, while in situations with low MRSA transmission rates omission of screening may be favorable.

**Conclusion:**

Early detection of MRSA by rapid PoC or PCR technologies and consistent implementation of appropriate hygienic measures lead to high economic efficiency of MRSA management. Whether general or targeted screening is more efficient depends mainly on epidemiological and infrastructural parameters.

## Background

Infections with Methicillin-resistant *Staphylococcus aureus* (MRSA) cause heavy financial burden on healthcare systems worldwide [1–3]. In particular, hospitals are highly economically affected by the consequences [4]. About 5 % of all hospital-acquired infections are caused by MRSA [5]. Main cost drivers are prolongation of hospital stay and contact precaution measures [6]. While the economic impact on nosocomial MRSA acquisition is controversial, it has been proven that rapid and precise identification of MRSA carriage at hospital admission reduces turn-around-time and thus the number of days in precautionary isolation [7, 8].

Health care providers have to decide which screening strategy to follow and which diagnostic technology to use in consideration of medical and economic criteria. The three main strategic options are universal screening, targeted risk-based screening or the omission of screening. Polymerase-Chain-Reaction (PCR), culture and Point-of-Care (PoC) methods are the diagnostic technologies available on the market [9].

Several screening strategies have been studied in terms of their cost-effectiveness [10–12]. However, little is known about the impact of the concrete implementation of a diagnostic test system in an MRSA hygiene management of a hospital. Aim of this study was to determine which MRSA screening and management strategy causes the lowest expected cost for a hospital with special focus on PoC technology, a relatively new technology that is not yet widely used for MRSA screening management.

## Methods

### Analytical model

**Fig. 1:**
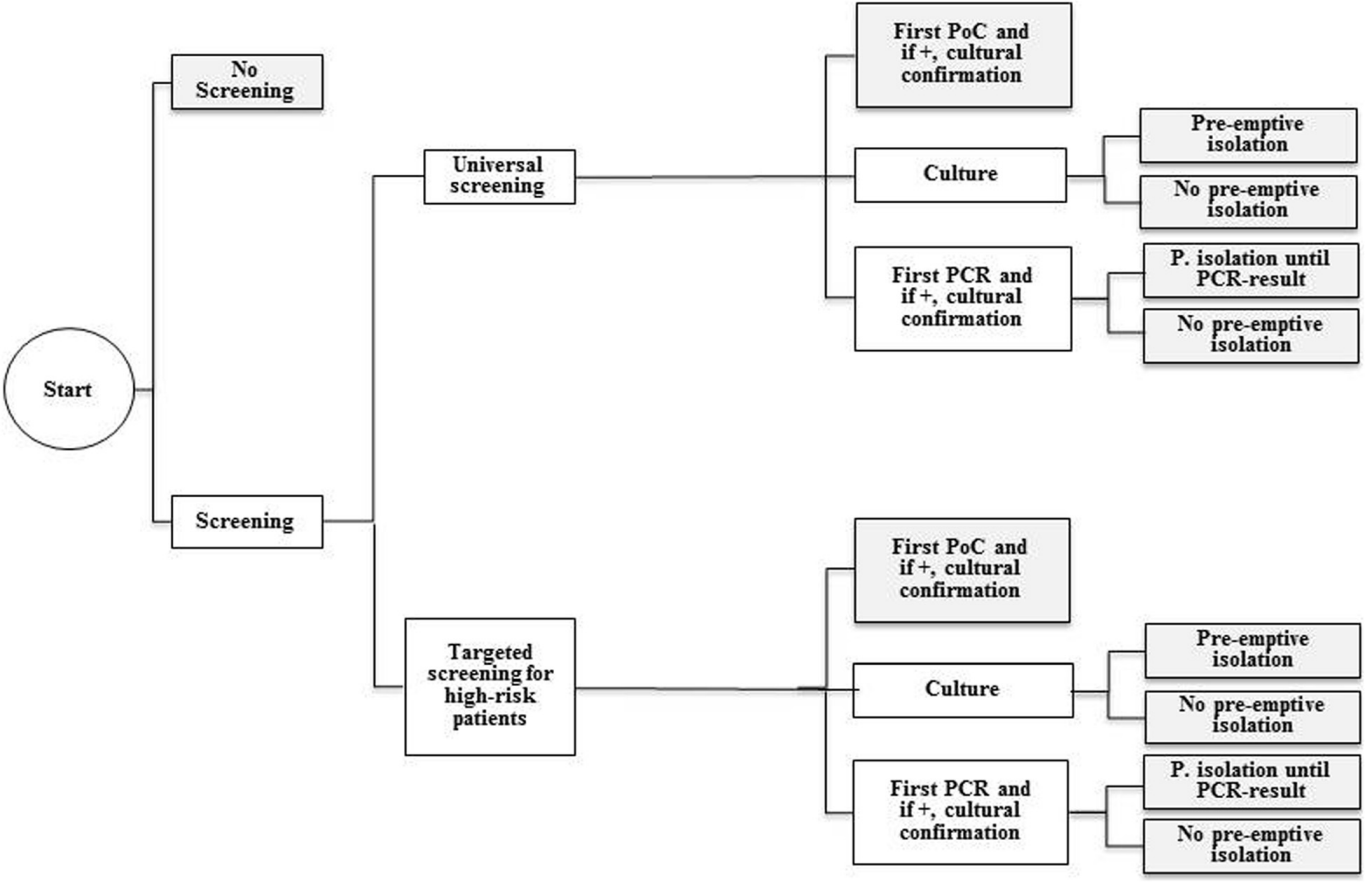
Model structure of the decision tree: 4-step-approach with decisions to (1) screening or not screening, (2) universal or targeted screening of high risk patients, (3) PoC, PCR or culture test and (4) pre-emptive isolation or no pre-emptive isolation

The analysis is based on a multi-stage decision model that assesses the expected cost of alternative MRSA admission screening and infection control management strategies. The decision tree developed for this purpose is shown in Fig. 1. The first branch is the decision between “no screening” and “screening”. Here, the hospital has to decide whether a screening strategy will be implemented or not. If a screening is conducted, it must be decided whether universal or targeted screening should be applied. Universal screening means that all patients admitted to the hospital are screened for MRSA. Targeted screening includes only high risk patients. In Germany, the Commission for Hospital Hygiene and Infection Prevention (KRINKO) gives recommendations on risk factors (e.g., known MRSA patient history, previous hospitalization in the last 12 months, contact with animal farms, chronic care, previous antibiotic therapy in the last 6 months) [13]. According to these recommendations, health facilities can define additional factors based on individual risk assessment. Further branches model the chosen test method (PoC, PCR or culture methods) and whether patients are taken under isolation while waiting for test results (pre-emptive isolation).

Finally the following 11 strategies were derived:

- no screening,
- universal screening with PoC testing (cultural confirmation of positive test results),
- universal screening with cultural testing and pre-emptive isolation,
- universal screening with cultural testing and without pre-emptive isolation,
- universal screening with PCR testing (cultural confirmation of positive test results) and pre-emptive isolation until PCR result,
- universal screening with PCR testing (cultural confirmation of positive test results) and without pre-emptive isolation,
- targeted screening of high risk patients with PoC testing (cultural confirmation of positive test results),
- targeted screening of high risk patients with cultural testing and pre-emptive isolation,
- targeted screening of high risk patients with cultural testing and without pre-emptive isolation,
- targeted screening of high risk patients with PCR testing (cultural confirmation of positive test results) and pre-emptive isolation until PCR result,
- targeted screening of high risk patients with PCR testing (cultural confirmation of positive test results) and without pre-emptive isolation.

### Input parameter

As shown in Table 1, most data were taken from a previous literature review conducted by our working group [14]. Values regarding the cross-transmission rates have been updated by reassessment of the literature: RT_no iso_ (new: 0.0443, old: 0.01), RT_iso_ (new: 0.0033, old: 0.0029). The data of two more studies [15, 16] have been incorporated, as they are examples of higher cross-transmission rates, especially in situation without isolation, which were previously in [14] under-represented. Other data are based on in-house references of the University Medicine of Greifswald (UMG) and Mölnlycke Health Care.

**Table 1:** Definition and quantification of input parameter

Parameter	Description	Value	Reference
*P*_*t*_	MRSA prevalence of all inpatients	3.075 %	[14]
*P*_*r*_	MRSA prevalence of high-risk inpatients	11.94 %	[14]
*P*_*n. i*._	MRSA prevalence of patients without indication for a targeted screening	2.09 %	Calculation based on in-house data (UMG)^a^
*RT*_*no iso*_	Rate of MRSA transmission not in isolation per day	0.0443	[14], updated with [15, 16]
*RT*_*iso*_	Rate of MRSA transmission in isolation per day	0.0033	[14], updated with [15, 16]
*Sen*_*PoC*_	Sensitivity of PoC method	95 %	In-house data (mölnlycke hc)
*Sen*_*PCR*_	Sensitivity of PCR method	91.09 %	[14]
*Sen*_*cul*_	Sensitivity of culture method	88.73 %	[14]
*SP*_*PoC*_	Specificity of PoC method	97.5 %	In-house data (mölnlycke hc)
*SP*_*PCR*_	Specificity of PCR method	95.79 %	[14]
*SP*_*cul*_	Specificity of culture method	93.23 %	[14]
*C*_*pre iso*_	Average costs for pre-emptive isolation per day	62.77 €	[14]
*C*_*MRSA*_	Average costs per MRSA case per day	506.92 €	[14]
∅ *LOS*_*MRSA*_	Average length of stay of MRSA patients in days	24.88	[14]
*T*_*PoC*_	Turn-around time of PoC method in days	0.01389	[9]
*T*_*PCR*_	Turn-around time of PCR method in days	0.29	In-house data (UMG)
*T*_*cul*_	Turn-around time of culture method in days	2.5	In-house data (UMG)
*C*_*PoC*_	Costs for a single PoC test	30 €	[9]
*C*_*PCR*_	Costs for a single PCR test	20.50 €	[14]
*C*_*cul pos*_	Costs for a single culture test with positive result	24.10 €	[14]
*C*_*cul neg*_	Costs for a single culture test with negative result	6.40 €	[14]
*C*_*f*_	Costs for follow-up screening	6.40 €	Costs of a negative culture test are assumed
*C*_*c*_	Costs for screening a contact patient	6.40 €	Costs of a negative culture test are assumed
*Pat*_*c*_	Number of contact patients	2	calculation based on in-house data (UMG)
∅ *LOS*_*reg*_	Average length of stay of regular patients in days	8	[14]
*Pat*_*t*_	Number of total inpatients per year	35,322	In-house data (UMG)
*Pat*_*risk*_	Number of high-risk patients per year	3,532	In-house data (UMG)
*Pat*_*n. i*._	Number of patients without indication for a targeted screening per year	31,790	In-house data (UMG)

^a^ *UMG* University Medicine Greifswald

### Assumptions

For the analytical model, several assumptions have been made:

1. Positive MRSA test results by PCR or PoC but must be confirmed by a culture test.
2. Negative MRSA test results by PCR or PoC will not be confirmed by a culture test.
3. If the MRSA test is negative, pre-emptive isolation is immediately discontinued and no further steps are taken.
4. Due to the short turn-around-time of the PoC test no pre-emptive isolation is taken into account.
5. Decision tree branches end after MRSA is excluded or after MRSA eradication treatment and follow-up screening has been conducted.
6. Cultural test method is applied for follow-up and contact-patient screening. These test results assumed to be negative.
7. Secondary MRSA cases (due to transmission events from index patients) are included in the analysis and causes costs of MRSA infections.
8. Tertiary MRSA cases (due to transmission events from secondary patients) are not included in the analysis.
9. Screening of contact persons is only necessary if no pre-emptive isolation of MRSA patient is conducted.

### Cost calculation

Expected costs for each screening strategy are calculated in the decision model. Calculations are based on the values of input parameters stated in Table 1. The principle is demonstrated exemplary for targeted PCR screening with pre-emptive isolation measures by the following formulas of Table 2.

**Table 2:** Calculation of expected costs in decision model (using the example of strategy “targeted PCR screening with pre-emptive isolation measures”)

Combinations of true and tested MRSA status of screened patients	Costs per patient*:	Number of patients*:
MRSA positive	*C*_*PCR*_ *+ C*_*culpos*_ *+ C*_*preiso*_ * (T_*PCR*_ *+ T*_*cul*_ *) + C*_*MRSA*_ * (Ø LOS_*MRSA*_ *- T*_*PCR*_ *-T*_*cul*_ *) + C*_*c*_ *Pat_*c*_ *+ Ø LOS*_*MRSA*_ * RT_*iso*_ * C_*MRSA*_ * Ø LOS_*MRSA*_	*Pat*_*risk*_ * P_*risk*_ * Sen_*PCR*_ * Sen_*cul*_
PCR (+) and culture (+) → true positive		
MRSA negative	*C*_*PCR*_ *+ C*_*culpos*_ *+ C*_*preiso*_ * (T_*PCR*_ *+ T*_*cul*_ *) + C*_*MRSA*_ * (Ø LOS_*MRSA*_ *- T*_*PCR*_ *-T*_*cul*_ *) + C*_*c*_ *Pat_*c*_	*Pat*_*risk*_ * (1 - P_*risk*_ ) * (1 - SP_*PCR*_ ) * (1 - SP_*cul*_ *)*
but PCR (+) and culture (+) → false positive		
MRSA positive	*C*_*PCR*_ *+ C*_*culneg*_ *+ C*_*preiso*_ * (T_*PCR*_ *+ T*_*cul*_ *) + [(Ø LOS*_*MRSA*_ *- T*_*PCR*_ *-T*_*cul*_ ) * RT_*noiso*_ *+ (T*_*PCR*_ *+ T*_*cul*_ ) * RT_*iso*_ ] * C_*MRSA*_ * Ø LOS_*MRSA*_	*Pat*_*risk*_ * P_*risk*_ * Sen_*PCR*_ * (1 - Sen_*cul*_ *)*
PCR (+), no confirmation because culture (-) → false negative		
MRSA negative	*C*_*PCR*_ *+ C*_*culneg*_ *+ C*_*preiso*_ * (T_*PCR*_ *+ T*_*cul*_ *)*	*Pat*_*risk*_ * (1 - P_*risk*_ ) * (1 - SP_*PCR*_ ) * SP_*cul*_
PCR (+), no confirmation because culture (-) → false positive		
MRSA positive	*C*_*PCR*_ *+ C*_*preiso*_ * T_*PCR*_ *+ [(Ø LOS*_*MRSA*_ *- T*_*PCR*_ ) * RT_*noiso*_ * + T_*PCR*_ * RT_*iso*_ ] * C_*MRSA*_ * Ø LOS_*MRSA*_	*Pat*_*risk*_ * P_*risk*_ * (1 - Sen_*PCR*_ *)*
but PCR (-), no culture test conducted → false negative		
MRSA negative	*C*_*PCR*_ *+ C*_*preiso*_ * T_*PCR*_	*Pat*_*risk*_ * (1 - P_*risk*_ ) * SP_*PCR*_
PCR (-), no culture rest conducted → true negative		
No high-risk patients (screening is not indicate)	Cost due to transmission (of MRSA-Patients, who were not screened)	Number of patients
MRSA positive	*Ø LOS*_*MRSA*_ * RT_*noiso*_ * C_*MRSA*_ * Ø LOS_*MRSA*_	*Pat*_*n.i.*_ * P_*n.i.*_
MRSA negative	*no costs*	*Pat*_*n.i.*_ * (1-P_*n.i.*_ *)*

*note: Variables are explained in Table 1

Additionally, cost differences of each strategy to the counterfactual strategy "no screening" were determined per admission to compare all screening strategies. Advantageous screening strategies resulted in cost savings (positive net benefit), unfavorable screening strategies in additional costs.

### Sensitivity analyses

Univariate sensitivity analyses of the parameters “Rate of nosocomial MRSA transmission” and “MRSA prevalence” were conducted. This allows simulating scenarios of hospitals with various structure and patient profiles (primary care vs. specialist care), departmental analyses (ICUs vs. general wards) or the consideration of MRSA outbreak situations.

MRSA transmission rate not in isolation was varied between 0.001 and 0.121 transmission per day. A division of each varied value by the factor 13 determined the corresponding values of MRSA transmission rate in isolation. This ratio of 1:13 resulted as the average of all included references (Table 1).

With regard to MRSA prevalence, an interrelation between the parameters “MRSA prevalence of all inpatients” and “MRSA prevalence of high-risk inpatients” is implied. Therefore, three scenarios at different MRSA prevalences (all inpatients/high-risk inpatients) were tested: low prevalence (0.5 %/3.5 %), middle prevalence (3.08 %/11.94 %) and high prevalence (7.5 %/25 %).

## Results

### Basic analysis

Targeted MRSA screening and hygiene management regimes using PoC or PCR technologies proved to be the most cost minimizing strategies in basic analysis. Average costs of all three methods were calculated between 468 and 470 € per admission. Strictly, risk-based (targeted) PoC screening showed the highest cost saving with a net benefit of 14.98 € per admission compared to “no screening”. However, deviations between the different screening regimes were quite moderate with the exception of universal cultural test methods. Cultural testing in combination with or without pre-emptive isolation caused immensely higher costs per admission compared to the omission of any MRSA-screening with additional costs of 264.40 € (no pre-emptive isolation) or 449.81 € (pre-emptive isolation) per admission, respectively (Table 3).

**Table 3:** Results of basic analysis for all 11 screening strategies: average costs per admission and cost savings (net benefits) in comparison to strategy “no screening”

Screening strategy	Average cost per admission	Cost-saving to "no screening"^a^
PoC (target)	468.14 €	−14.98 €
PCR (target, no isolation)	469.30 €	−13.82 €
PCR (target, isolation)	469.80 €	−13.32 €
Culture (target, no isolation)	501.66 €	18.54 €
Culture (target, isolation)	506.90 €	23.78 €
No screening	483.12 €	0.00 €
PoC (universal)	478.18 €	−4.94 €
PCR (universal, no isolation)	478.75 €	−4.37 €
PCR (universal, isolation)	500.07 €	16.95 €
Culture (universal, no isolation)	741.36 €	258.24 €
Culture (universal, isolation)	925.93 €	442.81 €

^a^A negative value indicates a positive net benefit

### Sensitivity analyses

#### Rate of MRSA transmission

**Fig. 2:**
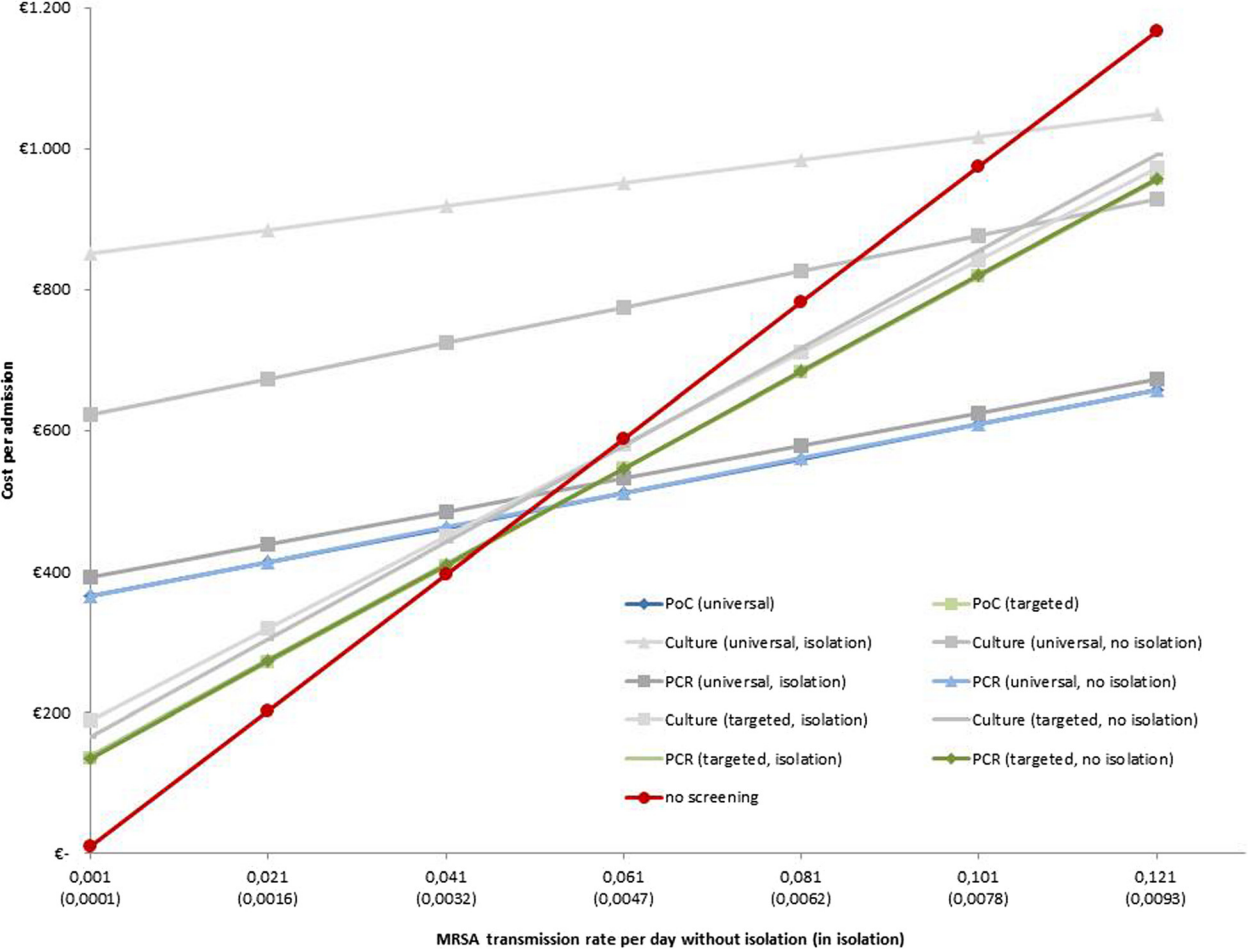
Sensitivity analysis: Graph of average cost per admission at different rates of MRSA transmission per day without isolation from 0.001 to 0.121 (corresponding rates of MRSA transmission per day in isolation from 0.0001 to 0.0093)

Curves representing the cost of the different screening regimes independence rates of nosocomial MRSA transmission not in isolation between 0.001 and 0.121 per day are shown in Fig. 2. The average cost per admission rises continuously with the increase of the MRSA transmission rate in all screening strategies. Three intervals depending on nosocomial MRSA transmission rate can be distinguished: Below a transmission rate of 0.04322 transmissions per day the omission of any screening method is the least costly strategy. Rapid (PoC or PCR) targeted screening regimens are most advantageous between a rate of 0.04322 and 0.05038 transmissions per day. This span corresponds to the range of MRSA transmission rates of normal wards in hospitals in Central Europe [17]. Universal rapid screening regimens conducted by PoC or PCR cause the lowest cost for a hospital above a transmission rate of 0.05038. This range can be assigned to high-risk areas in hospitals such as intensive care units or to outbreak situations [15, 18].

#### MRSA prevalence

**Fig. 3:**
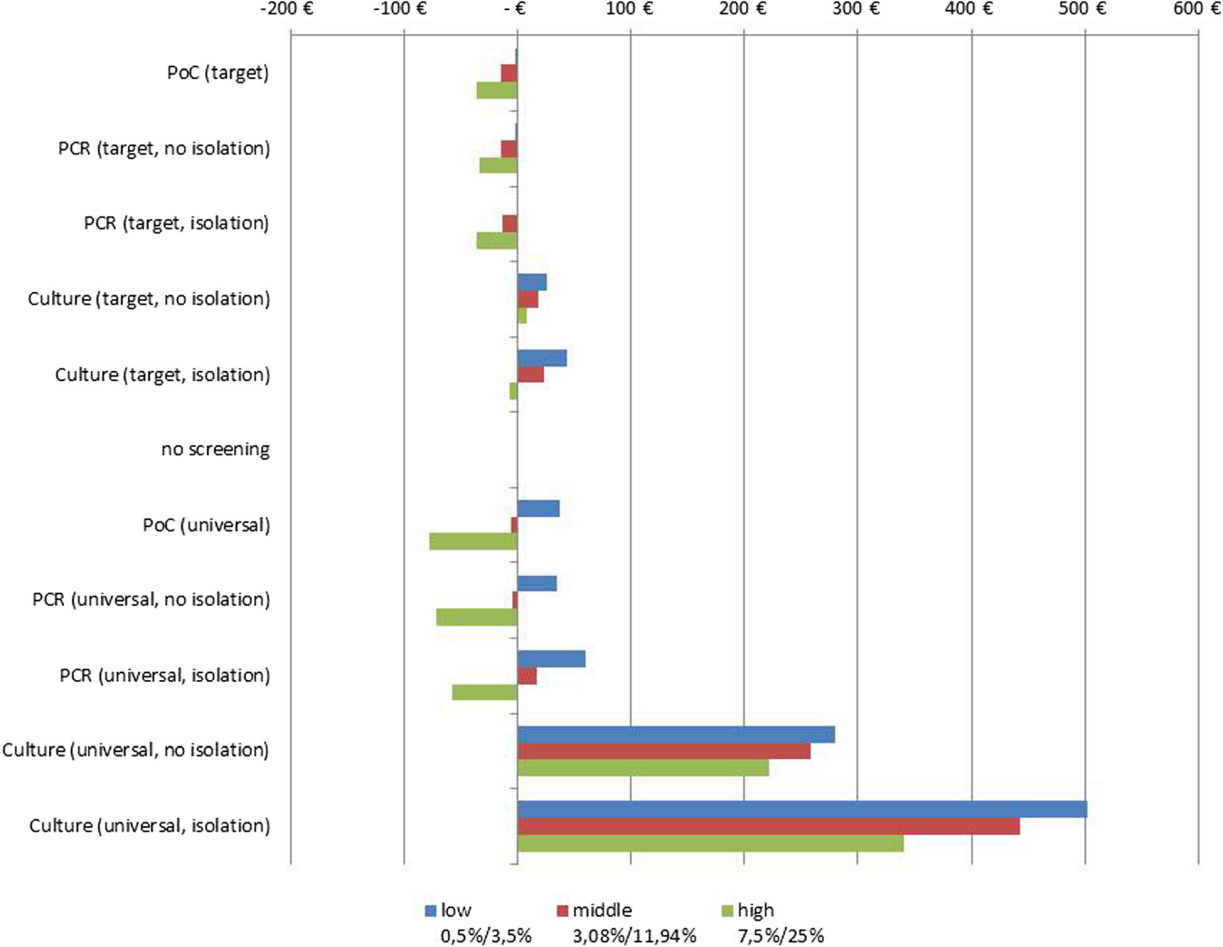
Scenario analysis: Column chart of average cost savings per admission in comparison to strategy “no screening” at 3 different MRSA prevalence scenarios (first value indicates prevalence of all patients, second value indicates prevalence of high risk patients)

Differences in the favorability of the specific diagnostic technologies for the three scenarios with low, medium and high MRSA prevalence are shown in Fig. 3. While at a low MRSA prevalence only rapid targeted screening strategies were beneficial versus “no screening”, ever more regimes became advantageous with increasing MRSA prevalence. At a high MRSA prevalence, 7 of 10 strategies are favorable. Here, “universal PoC screening” showed the highest cost savings with 77.22 € per admission compared to “no screening”, followed by the two universal PCR methods (with and without pre-emptive isolation measures).

## Discussion

The study shows a decision tree analysis to evaluate various MRSA admission screening methods as part of hygiene management regimes in hospitals. To our best knowledge, Point-of-Care technology is included in such an analytic model for the first time. Our results determine that rapid test methods (PoC or PCR) are always cost-minimizing in comparison to culture methods. By contrast, the decision on a universal or a risk-based method or the omission of any screening is highly dependent on epidemiological and infrastructural conditions regarding healthcare providers.

The MRSA cross transmission rate per day has been found as a very influential parameter by the sensitivity analysis. Based on the literature review of Tübbicke et al. [14] the basic analysis was calculated with an average transmission rate of 0.0443 transmissions per day. This coincides with the mid-interval of our sensitivity analysis (Fig. 3), in which the selective PoC strategy is most cost effective. However, the literature analysis shows outliers to both sides. For example, Raboud et al. [19] declared a very low rate of 0.00137 transmissions per day, while Jernignan et al. [18] showed a significantly higher rate with 0.140 transmissions per day. The latter study has been conducted with the particular patient clientele of neonatology. Other studies with a high cross transmission rate analyse outbreaks of MRSA [15, 16, 18]. Our study results demonstrate that in defined hospital departments (e.g., neonatology, ICUs, oncology, transfusion medicine, burn units) or in outbreak situations universal screening is not only medically but also economically viable.

Variations of MRSA prevalence have also strongly influenced test results. An increase of the MRSA prevalence tends to favor universal screening strategies, while in situations at lower prevalence (basic analysis) targeted risk-based methods are preferable. In line with literature, this underlines the regional epidemiology as an important decision [10]. In most of these studies, authors concluded risk-based screening to be cost-effective. Creamer et al. proved their statement in a backward stepwise logistic regression model by identifying defined risk factors [20]. Bühlmann et al. justified the restricted use of PCR methods with high costs and thus limited the implementation of pre-emptive isolation measures to high risk patients [21]. However, they also acknowledged that it might be different in settings with higher MRSA prevalence and transmission rates as shown in our sensitivity analyses. Shenoy et al. concluded from their randomized controlled trial that active PCR screening leads to a rapid discontinuation of contact precautions for MRSA, which can alleviate capacity constraints [22]. At the same time the authors also stressed that the net benefit of a screening program might depend on institutional characteristics including MRSA prevalence.

Our analysis also illustrates that the price of the test systems is not decisive. In contrast, the admission screening must be evaluated as part of all hygienic processes of a hospital. Early detection of MRSA by PoC or PCR diagnostics with their short turn-around-times and the consistent implementation of appropriate measures result in a high economic efficiency of MRSA management. Consequently, screening strategies with the cheaper culture methods prove comparatively unfavorable due to long turn-around times.

Our study has several limitations. First, the data quality needs to be critically examined. Most data was based on published international studies collected by a systematic literature research. For parameter where no sufficient information was found in the literature, we supplemented in-house data or made assumptions in consultation with hygiene experts. No probabilistic multivariate sensitivity analysis was performed. Instead, robustness of test results was proven by univariate sensitivity analyses and pointed to the dependence of individual parameters. Our analysis includes only transmissions from index patients to secondary cases, while transmissions from secondary cases and so on were excluded, thus underestimating the costs of transmissions. On the other hand, we assumed that all secondary cases cause costs due to MRSA infection for the hospital. Again, this is a simplification that may overestimate costs of transmission.

We did not simulate the effect of pre-test factors that may influence the sensitivity of the screening, e.g., use of different swabs or multi-site swabbing. The literature demonstrates that additional MRSA screening at extra-nasal sites increases MRSA detection by one-third compared to nares screening alone [23]. However, most PCRs and PoCs are not validated for multi-site testing and KRINKO recommendations favor nasal screening. For this reason, we have limited our model on the nares screening.

Finally, the analysis was conducted from the perspective of the healthcare provider and assessed its expected costs. A macroeconomic analysis may come to different statements, since more cost and benefit effects of patients or health insurances must be included. An important preliminary work for this purpose is the computer simulation study by Lee et al., which determined the economic impact of the implementation of a universal MRSA surveillance from the societal and third party-payer perspectives [12]. The extension of the analytical model is a potential research question for future work, especially in terms of inter-institutional cooperation between different healthcare providers and the economic evaluation of pre-admission MRSA screening.

## Conclusion

In summary, we developed an analytical model as decision-making tool that allows recommendations for the implementation of a cost-effective MRSA admission screening and infection control management strategy. Obviously, the results demonstrate the economic advantageousness of modern MRSA diagnostic techniques, particularly the Point-of-Care technology. However, it is clear that there is no silver bullet of uniform screening management, but that the knowledge of individual epidemiological and infrastructure parameters of the whole hospital or specific areas is crucial.
